# Livestock Disease Management for Trading Across Different Regulatory Regimes

**DOI:** 10.1007/s10393-018-1312-y

**Published:** 2018-02-12

**Authors:** Andrew M. Bate, Glyn Jones, Adam Kleczkowski, Rebecca Naylor, Jon Timmis, Piran C. L. White, Julia Touza

**Affiliations:** 1 Environment Department, University of York, Wentworth Way, York, YO10 5NG UK; 2 The Food and Environment Research Agency (FERA), Sand Hutton, York, YO41 1LZ UK; 3 Department of Mathematics, University of Stirling, Stirling, FK9 4LA UK; 4 Department of Electronics, University of York, Heslington, York, YO10 5DD UK

**Keywords:** Externality, Endemic disease, Disease management, Co-operation, Livestock

## Abstract

The maintenance of livestock health depends on the combined actions of many different actors, both within and across different regulatory frameworks. Prior work recognised that private risk management choices have the ability to reduce the spread of infection to trading partners. We evaluate the efficiency of farmers’ alternative biosecurity choices in terms of their own-benefits from unilateral strategies and quantify the impact they may have in filtering the disease externality of trade. We use bovine viral diarrhoea (BVD) in England and Scotland as a case study, since this provides an example of a situation where contrasting strategies for BVD management occur between selling and purchasing farms. We use an agent-based bioeconomic model to assess the payoff dependence of farmers connected by trade but using different BVD management strategies. We compare three disease management actions: test-cull, test-cull with vaccination and vaccination alone. For a two-farm trading situation, all actions carried out by the selling farm provide substantial benefits to the purchasing farm in terms of disease avoided, with the greatest benefit resulting from test-culling with vaccination on the selling farm. Likewise, unilateral disease strategies by purchasers can be effective in reducing disease risks created through trade. We conclude that regulation needs to balance the trade-off between private gains from those bearing the disease management costs and the positive spillover effects on others.

## Introduction

Globalisation has led to increased movement of goods and commodities, including livestock and livestock products (Knobler et al. 2006). More frequent and wider-ranging movements of livestock and livestock products increases both (1) the risks of new diseases being introduced, resulting in a higher frequency of epidemics that can cause great economic, animal welfare and environmental harm (and if zoonotic, can threaten human health too) and (2) the movement of endemic diseases, making the management of such endemic diseases more difficult and costly (Perry et al. 2013; Daszak et al. 2000). Reducing the risk of disease and protecting livestock health requires coordinated actions at international and national levels, with animal trade posing a governance challenge as it can result in the introduction of pathogens to previously disease-free areas (e.g. Fèvre et al. 2006; Perrings et al. 2010; Thompson et al. 2016). The role of individual producers (Gunn et al. 2008) and the interaction of public and private interests (Hennessy and Wolf 2015) are therefore critical.

For livestock diseases, the risk of an outbreak and subsequent spread and control of a disease can be affected considerably by farming practice across trading partners (Leibler et al. 2009; Brennan and Christley 2012). However, the management of endemic diseases can be challenging and costly for livestock producers (Bennett 2003; Knight-Jones and Rushton 2013). Economic theory views the problem of spread of an infection as a form of biological pollution (Daszak et al. 2000; Horan et al. 2002), where an individual producer’s attempts to eradicate the disease on their farm is undermined by the likelihood that the disease will be reintroduced into the herd as a result of their neighbour’s herd becoming infected or through trade of infected animals. Conversely, a livestock producer’s investment in biosecurity measures can reduce disease risk and potential damages for neighbours and trading partners, leading to positive externalities for these other parties and the potential for free-riding on biosecurity (Hennessy and Wolf 2015). This is known as a filterable externality because a producer’s biosecurity choices filter the risk of disease infection and damages to others (Shogren and Crocker 1991; Reeling and Horan 2015, 2017). Thus, strategic alliances among producers may emerge as a result of these bilateral interactions (Hennessy et al. 2005; Hennessy 2007; Horan et al. 2015). More recently, Reeling and Horan (2015) have shown that where individual producers have a greater ability to secure the benefits from private actions to control their own risk, greater levels of biosecurity strategic relationships among producers and improved overall biosecurity are more likely to emerge.

Given that alternative disease management strategies of livestock disease can lead to different benefits and costs to individual farmers, more information is needed on those measures that can incentivise unilateral actions. Here, we estimate the private benefits of self-protection from disease management strategies and compare these to the benefits that can be obtained from spillover effects of the biosecurity actions of trading partners. We quantify the extent to which disease damages resulting from trading with an infected farm can be filtered by biosecurity actions carried out by the seller, or by unilateral actions carried out by the purchaser.

Our analysis is based on the illustrative case study of bovine viral diarrhoea (BVD) across Scotland and England. BVD is a disease of cattle that is endemic across the UK, Europe and much of the rest of the cattle-producing world that cases significant economic losses to the cattle industry (Greiser-Wilke et al. 2003). Approaches to the control of BVD are highly variable between countries, and in our illustrative example, the different actions of sellers and purchasers are a consequence of differing regulatory regimes in the two countries. Following industry pressure, Scotland has developed a control and eradication scheme for BVD within Scotland (Scottish Government 2016). This involves compulsory movement restrictions on cattle linked to annual testing and culling. In England, a BVD test-and-cull (test-cull) scheme has recently been introduced (BVDfree 2016), but this scheme is voluntary, i.e. there is no regulatory force and thus no compulsory movement restrictions behind it. Vaccination is also a common management policy for BVD, with nearly 80% of surveyed English cattle farmers saying they administer BVD vaccines to at least some of their cattle (Cresswell et al. 2014). Therefore, management of BVD in England may consist of doing nothing or various combinations of vaccination only, test-cull only and test-cull with vaccination.

BVD is normally a mild (transient) infection (TI) that lasts around 2–3 weeks leading to lifelong immunity. However, complications can arise during pregnancy including abortions, miscarriages, birth defects and in utero transmission resulting in a superinfectious persistently infected (PI) foetus (Baker 1990; Lanyon et al. 2014). When born, a PI calf will shed large quantities of BVD virus for all of its life, and its lifespan is usually reduced to around 6–18 months. Many PI cattle suffer from fatal mucosal disease, and PI cattle are usually ill-thrifty and slow growing. Testing the status of foetuses cannot be done reliably, meaning that PI foetuses can remain hidden in immune pregnant cows/heifers (colloquially known as ‘Trojan Cows’) until birth (Lanyon et al. 2014). BVD epidemics often burn out in small and closed farms, a phenomenon called ‘self-clearance’ by Lindberg and Houe (2005). However, few farms are closed; each year around 65% of beef herds and 55% of dairy herds in the UK purchase replacement breeding cattle (Gates 2013, p. 113), whereas Gates et al. (2013) found only 4% of Scottish beef suckler herds had no replacement cattle purchases over a 3-year period. Animals brought in through trade provide a pathway for the introduction of BVD to a naïve farm and a supply of susceptible cattle for infected herds.

This case study illustrates the need to understand the relative benefits to individual farmers of regulatory versus voluntary approaches to livestock disease control. There is often a reticence on the part of many governments to impose unilateral regulatory requirements that can be both expensive and politically undesirable, given that the maintenance of livestock health depends on the combined actions of many different countries. By estimating the benefits (i.e. reduced disease damages) and costs to farmers of management strategies under regulatory requirements, our work offers some insights on whether disease management strategies followed unilaterally produce good-enough outcomes for farmers who bear the associated biosecurity costs, as well as for their trading partners in reducing disease damages (i.e. filtering the externality), and thus their potential for limiting the damages of the disease beyond the initial importer.

## Methods

BVD depends greatly on the destiny of a handful of PI cattle within a herd. Such small numbers mean that each birth and death could be the difference between the disease persisting and ‘self-clearance’, and thus, the stochasticity around PI births, deaths, disease transmission, demographics, movement and management timing is important to understand the efficiency of different disease management strategies during a BVD epidemic. Consequently, we use a stochastic agent-based model, written in NetLogo (version 5.3.1), to model this bioeconomic problem. This model incorporates disease dynamics, cattle population dynamics and a discounted measure of day-to-day net benefits for the farm; the latter encompasses revenues from selling or culling cattle as well as disease-related costs and management costs for test-culling and vaccination. We consider three alternative disease management strategies: vaccination only, test-cull only and test-cull with vaccination, with an initial condition where a PI calf is born in an otherwise susceptible herd, as well as the conditions under which these regimes may have a greater impact. This is done by comparing the farm’s exponentially discounted daily net benefits over a 5-year period obtained from undertaking one particular disease management strategy with the net benefits arising from a ‘do nothing’ option. Thus, ‘discounted net gains’ from following a particular disease management strategy are equal to the difference between the monetary net benefits of investing in that management strategy compared with a ‘do nothing’ approach in the presence of a BVD outbreak. A time frame of 5 years was chosen to allow enough time for BVD to spread and damages be realised in the farms. The net gains from the particular strategies derived from the model are computed within the context of the total disease damages, i.e. the ability of control to avert these damages. Disease damages are calculated by comparing revenues between a situation with no BVD outbreak and one with a BVD outbreak but no disease management strategy in place. We refer to this difference as the ‘net gains’ from a no-disease scenario and use this as the basis for evaluating the relative effectiveness of alternative disease management strategies in terms of how they compare with the no-disease scenario in reducing disease damages.

In this section, we describe the key assumptions of the bioeconomic model, with explicit parameter values given in Table 1. For further details, an ODD protocol report (Overview, Design concepts and Details, a standardised method for agent-based models; Grimm et al. 2006, 2010) is given in Appendix 1.

**Table 1:** Summary of Parameter Values Used to Assess the Efficiency of BVD Control Strategies for Farmers’ Self-Protection and Reducing Spillovers to Trading Partners.

Number of breeders	60 [AHDB (2016), includes 10–15 replacement heifers]
Weaning age	250 days (Nix 2014)
Time between conceptions	390 days (Nix 2014; CHAWG 2014)
Length of pregnancy	280 days
Age at first conception	450 days
Age for culling breeders	2930 days [Gates (2013, Chapter 6), Nix (2014)]
Recovery from disease	12 days (Baker 1990; Cherry et al. 1998)
Disease transmissibility of PIs	0.015 per animal per day (default)
Disease transmissibility of TIs	0.001 per animal per day (default, set to 1/15 of above)
Early/Late pregnancy threshold	150 days (Sørensen et al. 1995; Cherry et al. 1998; Viet et al. 2004)
Abortion rate	50% (default)
Mortality rate of PIs	1/365 per day (Duffell and Harkness 1985; Cherry et al. 1998; Viet et al. 2004)
Revenue from culling a breeder (PI or old age)	£500 (Nix 2014)
Revenue from culling a PI calf	£0 (no real demand for veal in UK)
Revenue from selling a calf at weaning	£500 (Nix 2014)
Cost of TI	£0.50 per animal per day [Gunn et al. (2004), adjusted by increase in beef prices between 2002 and 2016]
Cost of PI	£1.50 per animal per day [Gunn et al. (2004), as above]
Cost of testing	£5 per tested animal (SAC 2016) (£25 for annual passed test)
Cost of vaccine	£5 per breeder per year (farmacy.co.uk, Accessed: 3^rd^ August 2016) (£300 for annual vaccination of herd)
Frequency of testing and vaccination	365 days
Vaccine efficiency in abortion and PI reduction	85% (Newcomer et al. 2015)
Trade rate	0.02 per day [from 5 (median) and 9.3 (mean) of purchased replacement cattle per year; Gates (2013), Chapter 6]
Discount rate	5% per annum

### Farm Dynamics

**Fig. 1:**
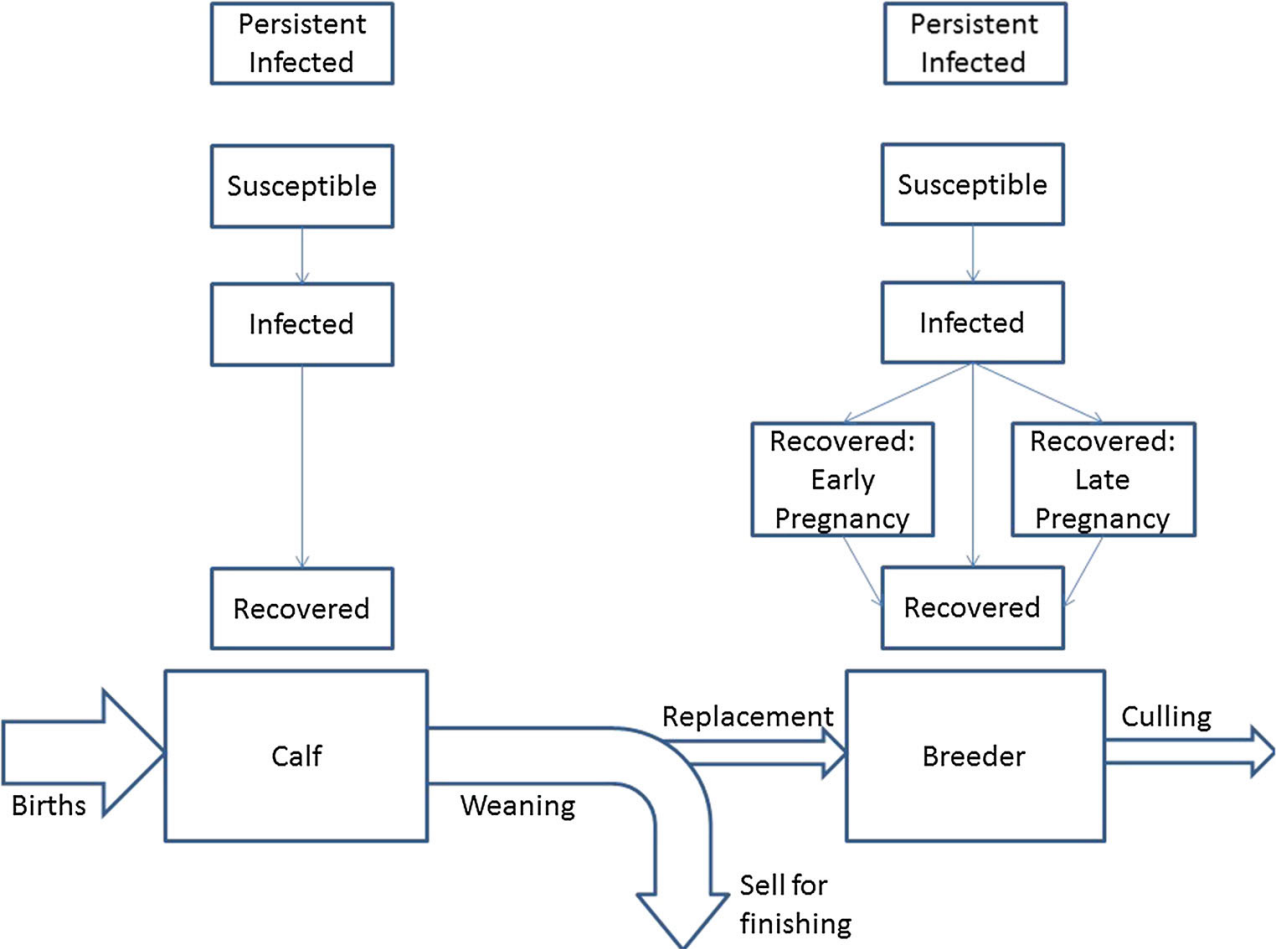
Phase diagram demonstrating the epidemiological and farm processes in a single closed farm.

Figure 1 shows the basic processes of a suckler beef farm. Cattle are split into 2 classes: calves and breeders. All newborn cattle up to weaning are categorised as calves. At weaning, the farmer chooses either to keep the calf as a breeder or sell it for finishing. Breeders remain on the farm until they are culled for old age. The decision to keep calves for breeding is modelled by keeping the breeding population constant by replacing culled breeders. In the model, this means that calves become breeders if there are fewer than the target number of 60 breeders at weaning (Table 1); otherwise, calves leave for finishing.

For simplicity, we assume all cattle are female and that artificial insemination is used. Likewise, we assume no mortality other than deaths related to BVD and those scheduled for culling; a reasonable approximation given that annual mortality is around 1–5% (CHAWG 2014; Nix 2014). We assume, in the absence of BVD, no abortions occur and that pregnancy leads to the birth of one calf. We ignore variable production costs since these costs are often considered on a per-breeder-calf pairing basis and these costs become constant under the constant breeder population. Lastly, when we consider trade between farms, we assume that trade timing is stochastic and consists of one breeder or calf (also chosen at random) accompanied by a payment from the purchasing farm to the selling farm. It is this movement that could lead to an outbreak in the purchasing farm if the moved animal is a PI, TI or Trojan cow.

### Disease Dynamics

Figure 1 demonstrates the structure of infection. The pathway of infection for an individual cow is from susceptible to infected to lifelong recovered. A transient infection lasts around 2–3 weeks and is latent for the first 5–7 days (Baker 1990). This means the period of infectiousness is between 7 and 16 days. Cherry et al. (1998) found that latent infection and colostral (mother’s milk) immunity had little effect on disease dynamics, so for the purposes of our model, we assume that transient infection lasts 12 days. We assume PI mortality is stochastic with the chance any PI dying each day being 1/365. Also, we assume that calves of PI breeders are also PI (Lindberg and Houe 2005).

For breeders, complications from infection during pregnancy need incorporating. Two more immune classes for infection during current pregnancy are added; one class for infection in the first 150 days of the current pregnancy and the other the last 130 days. We assume the former class results in either an abortion or a PI calf; whereas the latter leads to the birth of a lifelong immune calf. In both cases, the breeder moves to the usual lifelong immune stage at the end of the pregnancy. Other issues like birth defects are ignored or at least considered ‘abortions’ if culling is required. The probability of abortion varies in the literature, from around 40% (Viet et al. 2004; Sørensen et al. 1995) to 80% (Cherry et al. 1998). Given the importance of abortions and PI births to BVD epidemiology and costs, the sensitivity of the results to variation in abortion rate is examined in this paper.

We assume disease transmission occurs through ‘nose-to-nose’ direct contact of susceptible cattle with PI or TI cattle and that each animal within a farm can have such contact with any other. We ignore other routes of transmission like environmental and external sources of BVD. Following Cherry et al. (1998), we model this transmission as density-dependent. Other authors have used frequency-dependent transmission (Viet et al. 2004; Ezanno et al. 2007; Courcoul and Ezanno 2010; Gates et al. 2014). However, since the number of cattle does not change significantly during the epidemic (especially for the first few months), density-dependent transmission and frequency-dependent transmission are equivalent.

Estimates of the transmissibility of BVD vary across the literature. For example, Cherry et al. (1998) [using data from Houe and Meyling 1991) and Viet et al. (2004)] (and papers that follow: Ezanno et al. 2007; Courcoul and Ezanno 2010; Gates et al. 2014; Damman et al. 2015) differ in transmissibility parameters by a factor of around 6 (after density or frequency-dependent rescaling). Given this, we consider the sensitivity of the results to a range of different transmissibilities (ranging from 0.005 to 0.03 per animal per day). In line with others (Cherry et al. 1998; Viet et al. 2004), we assume that TIs are approximately 1/15th as infectious as PIs.

### Disease Control

The test-cull strategy we model is based around the regulatory regime in Scotland and the BVDfree scheme in England (Scottish Government 2016; BVDfree 2016). To implement this, the model includes an annual sampling test to the oldest five calves, costing £25 (Table 1). If two or more of these calves are found to be not susceptible, then the farm is considered to have the disease. If that is the case, all untested cattle are then tested and all PIs are culled; and for the next year (until the next annual test), all newborns are tested and culled if PI. If only one or no calves are found to be not susceptible in the annual test, then disease-free status remains and cattle can leave the farm without being tested.

With respect to movement, test-culling farms can purchase freely from other test-cullers, so there is mutual recognition of testing farms. However, test-culling farms purchasing from non-culling farms must test all purchased cattle and cull all PIs (at the testing purchaser’s expense). This means test-culling farms acquire BVD from farms that do not test-cull by purchasing Trojan cows, and not PI cattle. We assume tests have perfect sensitivity and specificity and that all tests and culls are instantaneous.

We assume vaccination does not prevent infection of the vaccinated cow, but does prevent infection of the foetus, leading to an 85% drop in abortions and PI calves (Table 1, we have ignored all other causes of abortions); thus, only breeders would be vaccinated.

### Initial Condition

The initial condition is the birth of a PI calf (and immune mother) with all other cattle susceptible on the farm. When we consider two farms, the PI calf is born in the selling farm, whereas the purchasing farm consists only of susceptible cattle. The ages of calves and breeders are uniformly distributed.

### Model Output

**Fig. 2:**
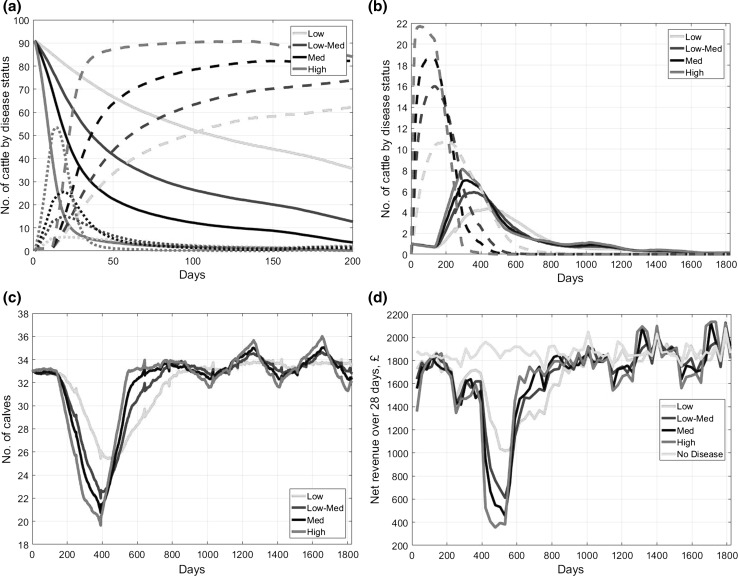
Time profiles for a single farm with no control under various disease transmissibilities (low 0.005, low-medium 0.01, medium 0.015, high 0.03) showing (**a**) number of susceptible (solid line), transiently infected (dotted line) and recovered (dashed line) cattle during the epidemic stage, (**b**) number of ‘recovered: early-pregnancy’ (dashed line) and persistently infecteds (PIs) (solid line), (**c**) number of calves and (**d**) net revenue over 28 days. The transmissibility values are those in Table 1. Sample of 500 for each parameter value.

For each day, the model computes the number of cattle by disease and age class as well as the daily profits (which includes revenues, disease-related costs and control costs). Figure 2 examines how these classes and profits vary over time following the birth of a PI calf. In essence, this enables us to understand the BVD epidemic and its consequences, establish how sensitive the model is to different abortion rates and transmissibility parameters and justify our choice for the default transmissibility parameter.

The results show the discounted net gains in a closed farm from adopting different management strategies (vaccination only, test-cull only and test-cull with vaccination; all fixed over time). Secondly, the results quantify how unilateral strategies either by the selling farm and the purchasing farm filter the externality of BVD from trade, i.e. reducing disease associated damages passed on through trade. This involves calculating the mean net gains of 100 simulations for each parameter-management combination.

## Results

### Dynamics of a BVD Outbreak on a Single Suckler Beef Farm

Figure 2a demonstrates that in the absence of control the disease spreads quickly through the herd, making most of the herd immune. For the case of high transmissibility, the infection has spread to almost all the herd within 1 month (at 40 days, about 90% of the herd is already immune). In contrast, for low transmissibility, the infection spreads much more slowly, with only about 40 immune cattle at 120 days.

Figure 2b demonstrates that as the disease spreads, the number of cattle infected during early pregnancy increases (dashed lines). These cases will later either result in abortion or the birth of a PI calf. This means a smaller delayed peak for PI (solid lines) occurs as the second generation of PI calves are born. This also leads to many abortions, resulting in a major decline in the number of calves (Fig. 2c). In all these figures, the peaks/troughs are flatter and wider for lower transmissibilities.

Figure 2d presents the monthly net revenues. In the first months, there are small disease-related losses from TI costs. This is followed by PIs being born a few months later, reducing the net revenues due to PI costs. However, the major drop in net revenues occurs even later when abortions (and dead PI calves) lead to a shortage in weaned calves for sale from day 380 onwards (depending on transmissibility parameter). It is therefore the lack of calves that provides the vast majority of the costs from BVD.

Overall, Fig. 2 shows that lower transmissibilities lead to shallower but longer-lasting peaks and troughs. These two factors (amount and duration of impact) largely cancel each other out, and the total costs increases by only 15–20% over the simulated period as a result of a sixfold increase in transmissibility. This suggests that transmissibility does not have a significant impact on the costs in the absence of controls. From now on we set medium transmissibility as the default (Table 1), since this is most consistent with the disease spreading through about 90% of the herd in 3–4 months (Houe et al. 1993; Moerman et al. 1993).

### Efficiency of Different Management Strategies in a Single Closed Farm

**Fig. 3:**
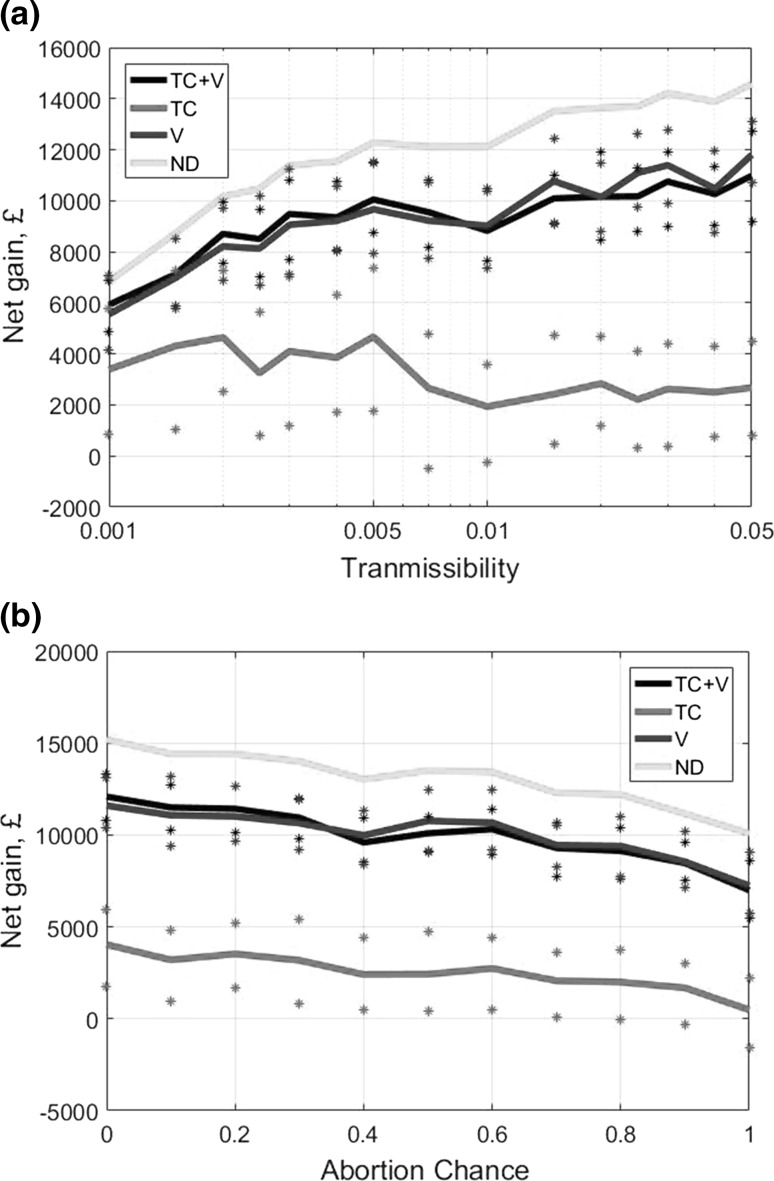
Discounted net gains for a single closed farm after 5 years following a BVD outbreak, under different disease management strategies while varying the parameters for (**a**) transmissibility and (**b**) abortion rate. Lines are means, and stars represent the upper and lower quartiles. Sample of 100 for each parameter value. *TC* test-culling, *V* vaccination, *TC* *+* *V* test-culling and vaccination, *ND* no disease.

Figure 3a shows the impact on a farm’s revenues from adopting alternative management strategies in the presence of a BVD outbreak over a 5-year period. The top line in Fig. 3a gives the full extent of damages of the epidemic if do nothing strategy is applied. For a default transmissibility of 0.015, this expected disease damages are around £15,000, with a vaccination strategy, for example, able to avert about 75% of these damages. Test-culling alone results in a net gain in farm revenues from applying this type of control across all transmissibilities compared with doing nothing. Its efficiency is lower for higher transmissibilities because in this case BVD damage occurs before annual testing and culling come into effect. In contrast, for lower transmissibilities, annual testing and culling intervention could happen in time to prevent further disease damages. However, results show that test-culling and vaccination together produce much larger gains than the test-cull strategy alone, with net gains of nearly £12,000 at default transmissibility. Vaccination alone produces a similar outcome, because vaccination prevents the vast majority of abortions and new PI calves, which are where the bulk of BVD damages occur.

Lower abortion rates are associated with higher damages (Fig. 3b). The expected impact of an abortion (where abortion rate equals 1) on disease damages is less than the expected impact from a PI calf birth (abortion rate equals 0) in a largely immune herd. Figure 3b also shows that test-culling has only a small net gain if abortion rates are high, but has a greater impact on reducing the impact on revenues of the disease for lower abortion rates. This is probably due to test-culling essentially increasing the ‘abortion’ rate to the value of one once detected. Vaccination alone and test-culling with vaccination both have a larger net gain across all abortion rates.

A corollary of lower damages for higher abortion rates (ND line in Fig. 3) is that even when the herd is largely immune, on average keeping a PI calf is more costly than culling at birth (which is equivalent to an abortion). This means that farmers that deliberately keep PI cattle to try and boost herd immunity have their own ‘vaccination’ cost without the reliability and security of normal vaccination (Fray et al. 2000).

### Impact of the Seller’s Management Strategy on the Purchasing Farm

**Fig. 4:**
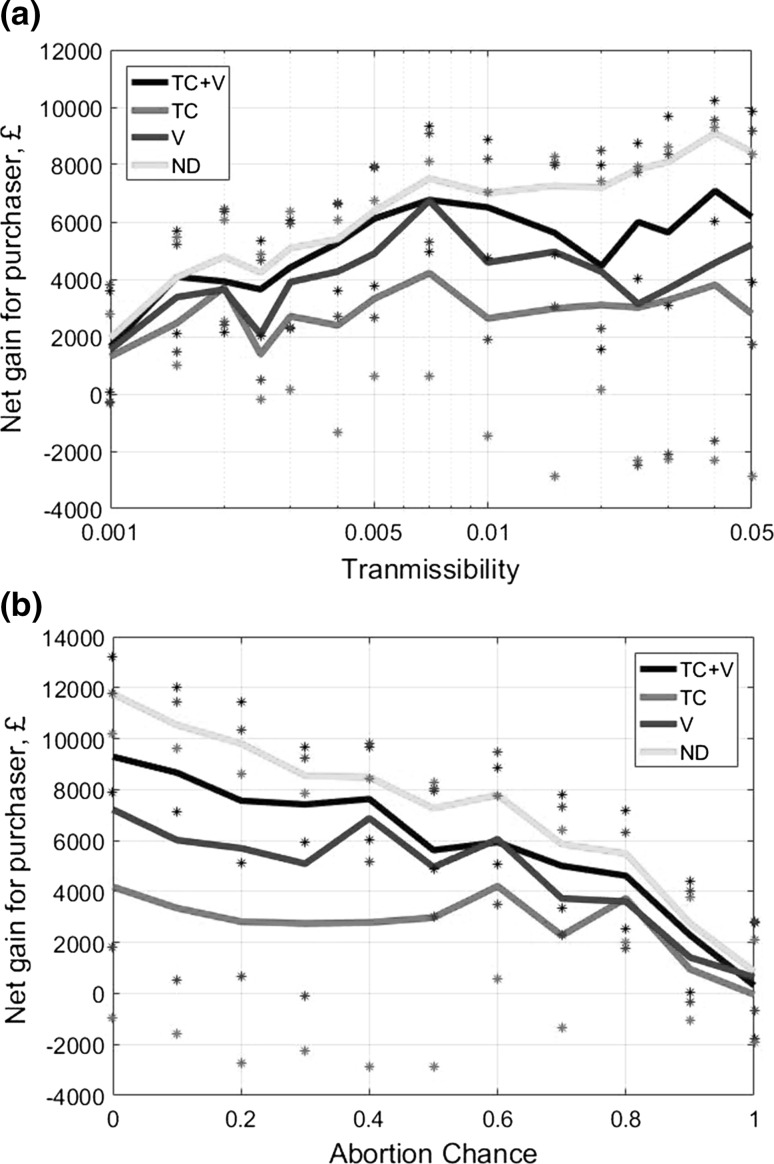
Discounted net gains for the purchasing farm after 5 years following a BVD outbreak when the trading partner (selling farm) undertakes biosecurity management strategies, while varying the parameters for (**a**) transmissibility and (**b**) abortion rate. The purchasing farm has no biosecurity control. Sample of 100 for each parameter value. *TC* test-culling, *V* vaccination, *TC* *+* *V* test-culling and vaccination, *ND* no disease.

Figure 4 covers the positive externality of BVD on the purchaser farm based on the different management controls in the seller. It therefore represents the impact of the seller’s alternative management options on the purchaser’s revenues. Figure 4a, b both shows that all potential disease management strategies carried out by the seller provide substantial gains to the purchaser’s revenues, i.e. result in filtering the externality. The net gains for the purchaser farm when the seller is under compulsory test-cull measures are lower than if the seller adopts a management regime that combines test-culling and vaccination. Thus, for the purchaser, the adoption by the seller of a strategy combining test-culling and vaccination is the next best option to a disease-free situation on the seller farm. This contrasts with the seller’s (weaker) private preference for only vaccinating in a situation without regulatory measures (Fig. 3). Note that the results have large interquartile ranges since the purchasing farm either gets little to no BVD or a full-blown BVD epidemic; there is not much in between.

Figure 4b demonstrates that abortion rate has an impact on the purchasing farm’s revenues, with overall BVD damages to the purchaser for buying cattle from an infected seller being low if the abortion rates are high. This is because high abortion rates lead to very few PI cattle and viable Trojan cows on the selling farm being passed on to the purchasing farm (in particular, a perfect abortion rate means the only PI is the first PI, which is very unlikely to be moved to the other farm).

However, note that Fig. 4 assumes that the purchasing farm does not vaccinate or test-cull. To further investigate this, Table 2 gives the benefits for the purchasing farm from each combination of management strategy in the selling and purchasing farms. Columns of Table 2 demonstrate that a purchasing farm would prefer buying from farms that both test-cull and vaccinate, independent of its own self-protected management strategy.

**Table 2:** Mean Net Gains for the Purchasing Farm Depending on Biosecurity Self-Protective Measures and Controls of the Trading Partner (Seller) Following Infection in the Selling Farm.

Seller	Purchaser			
	No control	Test-cull only	Vaccination only	Test-cull and vaccination
No control	£0	£1815	£4344	£5199
	(− £4721, £6131)	(− £3426, £6413)	(£2039, £6691)	(£2784, £7577)
Test-cull only	£2112	£2734	£5888	£6184
	(− £4006, £7530)	(− £2781, £7346)	(£3654, £8183)	(£4144, £8154)
Vaccination only	£5187	£5719	£6665	£6634
	(£2840, £9005)	(£4152, £8182)	(£4474, £8874)	(£5214, £8174)
Test-cull and vaccination	£5502	£6106	£6695	£7189
	(£4033, £8204)	(£4402, £9011)	(£5085, £8361)	(£5331, £9241)

Values are relative to the mean compared to a baseline when no control is applied. Interquartile range provided in brackets. Default parameter values used.

### Benefits Arising from Self-Protection in the Purchasing Farm

**Fig. 5:**
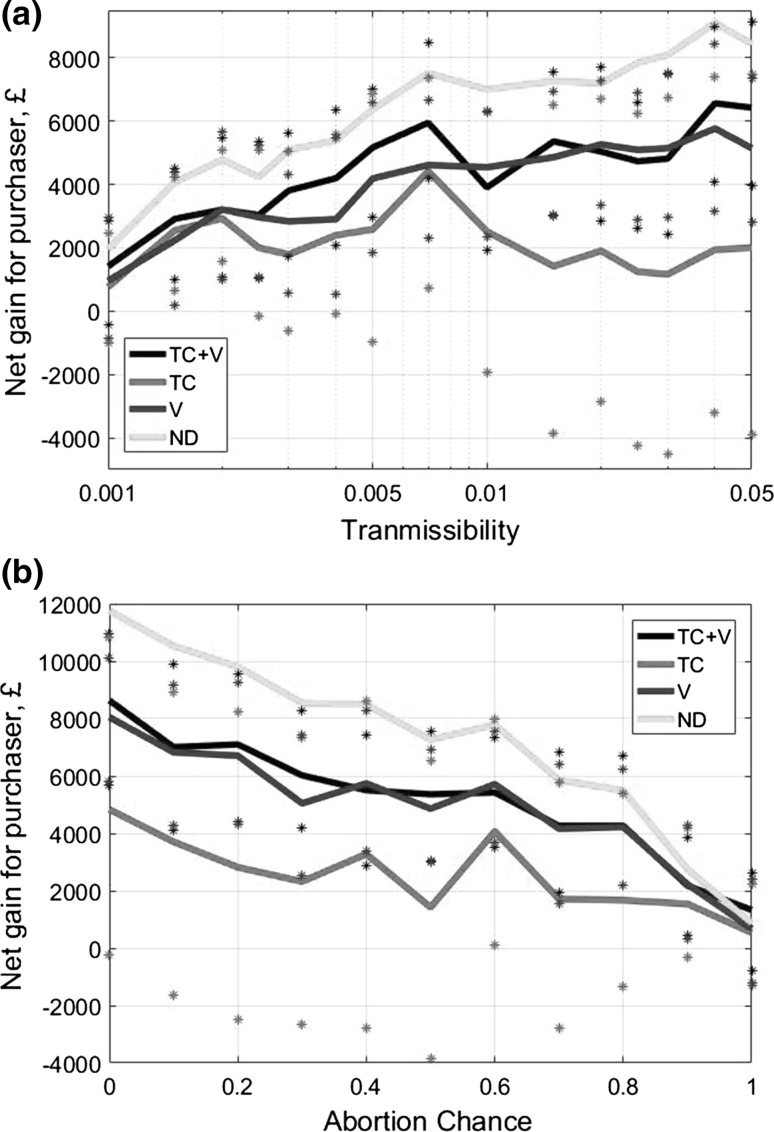
Discounted net gains for the purchasing farm after 5 years following a BVD outbreak when applies unilaterally self-protective disease management strategies, while varying the parameters for (**a**) transmissibility and (**b**) abortion rate. The selling farm has no biosecurity control. Sample of 100 for each parameter value. *TC* test-culling, *V* vaccination, *TC* *+* *V* test-culling and vaccination, *ND* no disease.

Figure 5 demonstrates that when a disease-infected selling farm is doing no control, the highest net gains for a unilateral strategy by the purchasing farm are achieved by conducting vaccination alone or test-culling combined with vaccination. For most parameter values, test-culling with vaccination is a slightly more effective strategy than vaccination alone, especially for lower abortion rates and transmissibilities. In addition, for lower transmissibilities, the test-cull strategy has a similar efficiency to the alternative strategies. Comparison of rows in Table 2 shows that vaccination with or without test-culling yields benefits to the purchasing farm, independent of the strategy of the selling farm. As before, there is a large variability in the simulation results leading to large interquartile ranges; the distribution of disease loads and hence losses and gains are all bimodal.

## Discussion and Conclusion

This study has set out to evaluate the knock-on effects of biosecurity actions of farmers on trading partners, particularly with respect to endemic diseases. Consistent with previous work, we demonstrate that diseases can cause externalities to trading partner in a way similar to biological pollution (Daszak et al. 2000). We additionally show that this pollution can be filtered by actions of the seller that lead to reduced risk of spreading the disease (Reeling and Horan 2017). However, we show that the management strategy that is best for the seller does not necessarily correspond to the strategy that best reduces the disease harm to the purchaser (comparing Figs. 3 and 4).

Our results emphasise that farms can at least partially protect themselves from acquiring the disease and/or reducing the damages by taking biosecurity actions unilaterally (i.e. even if their trading partners do not). Moreover, regulation can constrain the choices of biosecurity strategies available to individual farmers, impacting both the level of self-protection, and the spread of disease damages to others. Regulation needs to balance the trade-off between private gains from farmers own risk management and the positive knock-on effects their management has on others. Conversely, a lack of coordination between the actions of different farmers can generate significant disease damages and undermine efforts to control or eradicate endemic diseases (Shogren and Crocker 1991; Epanchin-Niell 2017).

In addition to these general results, our paper has specific policy implications for the control of BVD in the trade between England and Scotland. Regulations that enforce test-culling (like in Scotland) provide benefits to individual farmers experiencing a BVD outbreak by reduced disease damages. However, both vaccination alone and test-culling combined with vaccination are more efficient in averting disease damages compared with test-culling alone. These results therefore show that for farmers who are already vaccinating, a mandatory test-culling regulation does not seem to provide any additional private gains in reducing disease impacts on farms’ profits.

We also quantify the extent to which the disease damages by trading with a BVD-infected farm can be filtered by biosecurity actions carried out by the selling farm. Our results show that without regulatory restriction (i.e. England) the test-culling strategy is less effective in filtering the externality than vaccination alone, which is less effective than test-culling with vaccination. If test-culling is compulsory, as in Scotland, then the seller’s private best strategy would be to combine test-culling with vaccination, since this would result in significant reduction in disease damages to purchasers. Under a test-culling regulation strategy, private risk management incentives align with their ability to protect trading partners.

We also evaluated the effectiveness of unilateral actions carried out by purchasing farms. The best unilateral strategy to be implemented by a purchasing farm when trading with an infected selling farm is either test-culling and vaccination or vaccination alone, with the single most effective option depending on disease transmissibility and abortion rate.

Therefore, within the illustrative case of Scotland-England regimes, this paper shows that the test-culling regime enforced in Scotland provides private benefits for individual farmers in reducing disease damage from a BVD outbreak, but even larger reductions in the externality of trade imposed upon a purchasing farm. Thus, this strategy is particularly successful at reducing the spread and consequent damages of BVD to trading farms, i.e. reducing the spread of infection beyond an initial infected importer. However, test-culling is not the best strategy that a purchasing farm can conduct unilaterally to filter its damages from trading with farms that undertake no disease management. Moreover, the lack of compulsory regulation in England allows for farmers who neither test-cull nor vaccinate to cause significant damages to others, whatever the disease management strategy, including those that unilaterally undertake test-culling.

We have focused our analysis on the benefits of biosecurity under the condition that the disease is present. However, this approach neglects cases where no outbreak occurs, but disease management strategies are still applied and maintained. Our exploration of this case (Appendix 2) demonstrates that vaccination (with or without test-culling) results in positive expected net gains until the likelihood of an outbreak falls below around 10% for a closed farm. In contrast, test-culling alone has positive expected net gains until the likelihood of the outbreak falls below 3% and only has very small losses beyond this threshold value. As BVD starts to become rare, test-culling will become increasingly more effective and continue to provide net gains for farmers beyond the point at which vaccination is not economically worthwhile. Such changes in the effectiveness of different disease management strategies are important to consider when devising long-term livestock health strategies.

For generality and simplicity, we ignore potential seasonality of suckler beef farms. However, we suspect this results in an overestimation of disease-related damages as on a seasonal farm, births of PIs would correlate with other births and thus miss the season where most breeders are in early pregnancy. In addition, seasonality can affect trading patterns, in terms of cattle of particular ages and pregnancy status, which come with varying risks of spreading BVD (Gates et al. 2014). Another limitation of this study is that the price of cattle is independent of both the infection status and the farm’s biosecurity strategy [i.e. price endogeneity has been ignored, unlike Horan et al. (2015)]. However, as infection is often hidden or mild, only the ill-thrifty PIs would likely have a notable difference in price. Additionally, disease management status is not always public and this paper shows that purchaser could benefit from getting the information on the biosecurity measures applied by the seller. Armed with this information, the purchaser can benefit from choosing sellers who implement the best filtering strategies; this could lead to a price premium for those sellers who adopt the appropriate control strategies.

Finally, we have explored here that under appropriate conditions the benefits experienced by the purchaser as a result of biosecurity measures adopted by a seller may even exceed the private benefits these biosecurity measures bring to the seller. This opens new avenues of research in terms of assessing the efficiency of ex-border regulations to exporters.

## Appendix 1: ODD Protocol of Model

The model description follows the Overview, Design concepts, Details (ODD) protocol (Grimm et al. 2006, 2010).

### Overview

#### Purpose

This agent-based model of bovine viral diarrhoea (BVD) within a beef farm includes age structure, disease dynamics, control and disease dynamics. Given that BVD persists and is spread by a few PI individuals, an agent-based model with stochastic effects is important. The aim of this model is to establish the distribution of disease dynamics and economic cost given a variety of disease management strategies scenarios across two farms.

### Entities, State Variable and Scales

Each agent represents one bovid.

Time is discrete with time steps of 1 day. There is no spatial dimension; each farm is seen as a separate patch.

Each agent has several variables: (1) Age-group. Agents are either calves or breeders. (2) Age. Calves are between 0 and 250 days, whereas breeders are aged between 250 days and 2930 days. (3) Disease-stage, set as 0 (susceptible), 1 (transiently infected), 2 (recovered), 3 (persistently infected) and 4 (susceptible but vaccinated). (4) Disease age. This is a counter of days of how long an agent is transiently infected, starting at zero and at 12 days the agent recovers. (5) Pregnancy-age is a counter of days that determines when a breeder is pregnant, all breeders increase its pregnancy each day, and reset it to 0 when a calf is born at 365 days. (6) Early-pregnancy is a Boolean indicator that states whether the breeder became infected during the first 5 months (pregnancy-age between 0 and 150). During calving, this indicator is set to ‘false’. (7) Late-pregnancy is a Boolean indicator, like early-pregnancy, but is for the last 4 months of pregnancy (pregnancy-age between 150 and 280). During calving, this indicator is set to ‘false’. (8) Tested is a Boolean indicator for whether the agent has been tested before. (9) Farm gives the agent’s current farm.

### Process Overview and Scheduling

Following ‘Setup’, for each time step, the following processes are done in this order.

1. Disease-progress
2. Ageing
3. Death–births
4. Movement
5. Testing
6. Vaccination
7. Cashflow

### Design Concepts

#### Basic Principles

There are four main principles in this model. The first is the age/class structure. All newborns are calves, whereas breeders are culled when they are 2830 days old. When calves mature around the age of 250 days, they either become breeders or sold for finishing for £500. Farmers try to replace the breeders they have lost using weaned calves; consequently, calves become breeders when the farm has less than 60 breeders, whereas if the farm has 60 breeders, weaned calves are sold for finishing.

This second is disease dynamics. Susceptible cattle get infected (via density-dependent transmission) to become transiently infected. After 12 days, the infection goes and the cattle become immune. On top of this, there are persistently (lifelong) infected (PI) cattle. These are created in utero by the mother becoming infected during early pregnancy. If these cattle survive in the womb (abortions and birth complication are very common), the newborn calf is PI. These PIs are generally sicker than normal cattle and have an expected lifespan of 1 year, although many live much longer.

The third is control; test and cull and vaccination are control methods used by various farms. Test and cull is annual and will miss in utero PIs, whereas vaccination prevention infection for breeders.

The fourth is the two-farm structure with movement between the two farms.

##### Emergence

The disease dynamics and costs should depend on the use of controls but the nature of this is not known.

##### Adaptation

Agents do not have adaptive behaviour.

##### Objectives

Agents do not have objectives, although the farmer does.

##### Learning

Agents to not have adaptive behaviour. For simplicity, we assume the farmer does not either.

##### Prediction

No prediction by agents.

##### Sensing

No sensing.

##### Interaction

The only interaction between cattle is disease transmission.

##### Stochasticity

Transmission, PI mortality, abortions and movement are stochastic (via Bernoulli trials). Initial conditions have uniformly distributed ages with range depending on class. This age is converted to give the pregnancy age. The initial timing of annual tests and vaccination is a uniformly distributed (integer) between 0 and 364, inclusive. For two testing farms, we assume the annual tests are independent of each other, whereas we assume two vaccinating farms time their vaccinations together.

##### Collectives

There are no collectives other than those with given agent properties, e.g. breeders, infected.

##### Observation

The essential output is the daily cashflow of each farm. Additionally, the number of cattle by disease stage, the number of breeders infected early in the current pregnancy and the number of calves and breeders on each farm can be useful outputs.

### Details

#### Initialisation

The initial condition is the birth of PI from a Trojan cow in an otherwise susceptible beef farm. (The other farm is also naïve with no Trojan cows or PIs.) We create 60 breeders and 32 calves (the nearest integer to the long-term average of calves from numerous simulations) for each farm.

All of agents have early-pregnancy and late-pregnancy as false, but have random age uniformly distributed within the age range for calves/breeders. On top of this, all calves have pregnancy-age as −90, whereas all breeders have a pregnancy age that corresponds with their calving timings, i.e. for breeders younger than 730 days, set pregnancy-age as ‘age − 340’, between 730 and 1120, set pregnancy-age as ‘age − 730’.

For farms with vaccination, set the disease stage of all breeders old enough to be breeders during the last vaccination to disease-stage = 4, i.e. for breeders with age greater than 250 + vac-time, where vac-time is a number of days since last vaccination (between 0 and 364). This vac-time is the same on each farm. Test-time is the number of days since last annual test. Test-time is independent for each farm.

In the farm with the PI, the youngest calf has its age set to zero and disease-stage set to 3 (i.e. a newborn PI) and the breeder with lowest pregnancy-age that is at least 730 days old (i.e. had one pregnancy) has its pregnancy-age set to zero and disease-stage set to 2 (i.e. breeder has just given birth and is immune).

#### Input Data

There are no ‘Input data’.

#### Submodels

Setup combines the initialisation section with some visualisation instructions.

##### Disease-Progress

For each susceptible (disease-stage = 0 or disease-stage = 4) agent, take each transiently infected agent (with disease-age > 0, of the same farm) and get a random number between 0 and 1 and check if this number is less than the disease transmissibility of a TI. (This random number is uniformly distributed, and this applies to other random numbers.) If it is less than that, then set disease-stage of the susceptible agent to 1. Otherwise, take each PI agent and get a random number between 0 and 1 and check if this number is less than the disease transmissibility of a PI. If so, change the disease-stage of the susceptible agent to 1.

When setting the disease-stage to one, we need to check the pregnancy status for breeders and whether vaccination blocks this. For all breeders with disease-stage 1 and 15% of the time for breeders with disease-stage 4, we do the following: If pregnancy-age is between 260 and 390, set ‘late-pregnancy’ = true, and pregnancy-age 110 and 260, set ‘early-pregnancy’ = true, otherwise no change.

Following this, increase disease-age by one for all transiently infected agents (disease-stage = 1). Those that reach disease-age = 12 recover from this disease and become immune, i.e. set disease-stage = 2.

##### Ageing

It involves increasing the age of all agents by one, as well as increasing the pregnancy-age of all breeders by one. Following this, we check if calves have reached the age of weaning (250 days). At 250 days, the calf becomes a breeder if the total number of breeders on their farm is < 60 (i.e. becomes a replacement for recently culled breeder); otherwise, the calf is sold. The number of calves sold is recorded for profit calculation.

##### Death–Births

Firstly, check all PIs to see if they die from disease-related mortality. For each PI, get a random number between 0 and 1. If this number is less than 1/365 (i.e. expected lifespan of 1 year), then the agent dies. These deaths are counted according to their age structure so that these deaths can contribute to revenues/costs.

We also check for breeders reaching culling age of 2830 days. The number of breeder culls needs to be counted so that the revenue can be calculated.

Following the deaths we deal with births. Check all breeders and find those whose pregnancy-age of a breeder hits 390 days. For these breeders, of both early-pregnancy and late-pregnancy are false, a new susceptible (disease-stage = 0) calf is born. If late-pregnancy is true, a new immune (disease-stage = 2) calf is born. If early-pregnancy is true, then get a random (uniformly distributed) number between 0 and 1. If this number is less than prob-abort, then the pregnancy ends by abortion, stillborn or other complication. If this number is greater than prob-abort, a new PI calf is born. All calves are born on the same farm as their mother.

All the breeders that just gave birth reset the pregnancy-age to 0 and both early-pregnancy and late-pregnancy false.

##### Movement

Movement depends on the scenario. In this paper, we have two scenarios: 1-farm and 2-farm-1-way. For 1-farm, this section does nothing. For 2-farm-1-way, we consider moving cattle from farm 1 to farm 2.

If a random (uniformly distributed) number is between 0 and 1 compared with 0.02, then there is the chance of movement. If this is higher, nothing happens. Else, we will separate the case where either the seller tests but does not have disease-free status or the buying farm tests from other cases (since the former requires testing).

Pick a random agent from the selling farm. If the agent has not been previously tested and the selling farm does not test, then the buying farm pays for testing the agent. The agent tested status is changed to true and farm status changed to that of the buying farm. If this agent is PI, then it gets culled. Otherwise, just change the agents farm status to that of the buying farm.

An indicator that an agent has moved is used for the profit.

##### Testing

If test is set off (i.e. test = false) as a control method, then this section does nothing.

Otherwise, we increase the time since last test, test-time forward by one. If the farm’s disease-free status is false (i.e. recent outbreak) all newborn calves are tested and culled if PI. If test-time reaches the testfrequency (365 days), set test-time to 0 and make a test sample of the 5 oldest calves. If 2 or more of these are not susceptible, then test all untested cattle, cull all PIs and set the farm's disease-free status to false. Otherwise, set the farm's disease-free status to true. In all cases, set the test-status of all tested agents to true and count the tests and culls for costing reasons.

##### Vaccination

We start by increasing the time since last vaccination, vac-time forward by one. When this reaches 365, if the farm vaccinates, all susceptible breeders are vaccinated (i.e. disease-stage changed from 0 to 4), and if the farm does not vaccinate, all vaccinated breeders (like those purchased from another farm) lose protection (disease-stage changed from 4 to 0). Vac-time is reset to zero. We will assume both farms vac-time is the same on both farms.

##### Cashflow

If test = true and test-time = 0, then a test happened this turn, which gives a testTotalCost is testCost (a per-capita) times the total number of cattle in the farm. Other costs and revenues occur at a per-agent basis.

Discounting can be applied here too or applied to the cashflow output.

## Appendix 2: Management in a Single Farm When BVD is not Always Present

See Fig. 6.

**Fig. 6:**
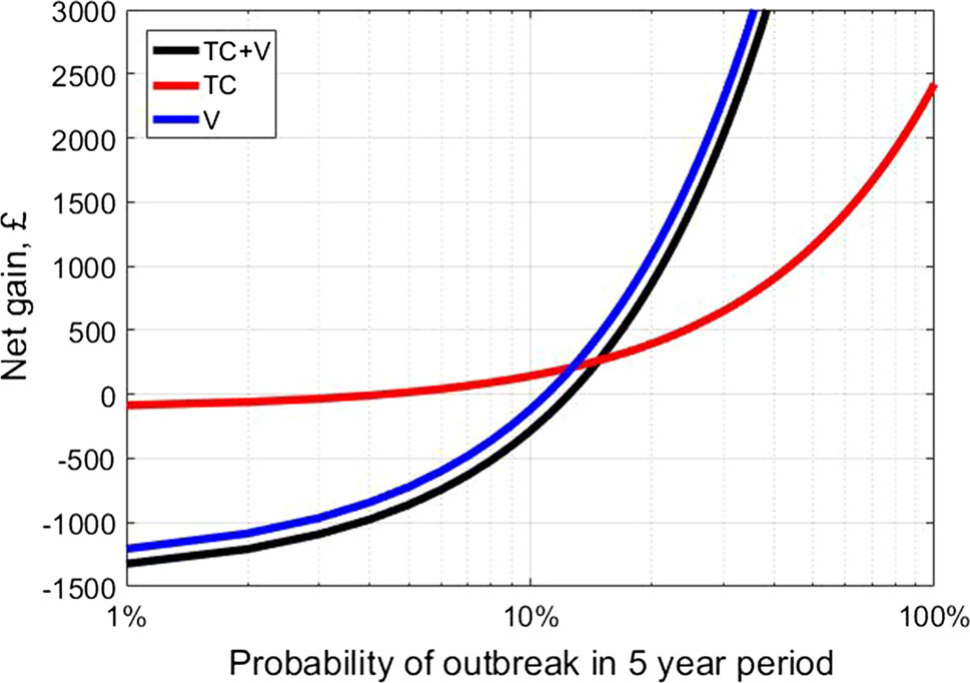
Discounted net gains for a single closed farm of undertaking biosecurity disease management strategies, given a probability of outbreak in a 5-year period. For the case where there is an outbreak, we use the mean net gain values from the default parameter sets in Fig. 3. If no outbreak occurs, the farm still incurs in an annual cost of £25 for testing and £300 for vaccination.
